# Neurocognitive Changes after Sustained Ketamine Administration in Children with Chronic Pain

**DOI:** 10.4172/2165-7386.1000215

**Published:** 2015-03-27

**Authors:** Amy Lee Bredlau, Brian T Harel, Michael P McDermott, Robert H Dworkin, David N Korones, James G Dolan, Heather R Adams

**Affiliations:** 1 Department of Pediatrics, University of Rochester, Rochester, New York, USA; 2 Cog State, Inc. Yale University School of Medicine, Child Study Center, New Haven, USA; 3 Department of Biostatistics and Computational Biology, University of Rochester, Rochester, New York, USA; 4 Departments of Anesthesiology and Neurology, University of Rochester, Rochester, New York, USA; 5 Departments of Pediatrics and Palliative Care, University of Rochester, Rochester, New York, USA; 6 Department of Public Health Sciences, University of Rochester, Rochester, New York, USA; 7 Department of Neurology Rochester, University of Rochester, New York, USA

**Keywords:** Pediatric, Neurologic, Memory, Cognitive function, Sedation

## Abstract

**Introduction:**

Ketamine has received attention recently as an agent for chronic pain. There are concerns, however, regarding the neurocognitive changes patients might experience after ketamine exposure.

**Methods:**

This prospective, uncontrolled study describes the neurocognitive functioning of 11 children with chronic pain before and after 2 weeks of daily oral ketamine exposure. Neurocognitive assessment was performed at baseline, Week 2, and Week 14. We hypothesized that there would be declines in neurocognitive scores at either Week 2 or Week 14.

**Results:**

No decline in neurocognitive function was detected in the children investigated. Mean scores for tests measuring executive function and memory were improved at Weeks 2 and 14 compared to baseline.

**Discussion:**

This study did not detect any decline in neurocognitive scores in a small number of children exposed to 2 weeks of oral ketamine therapy. Randomized, controlled studies of the neurocognitive effects of ketamine in children are recommended to further investigate these preliminary findings.

## Introduction

Ketamine is an agent that has been used as a dissociative anesthetic during painful procedures and during induction of anesthesia in both adults and children for greater than 50 years [1]. However, there are concerns of the long-term neurocognitive effects of ketamine administration. Children who have had multiple surgeries requiring anesthesia with (and without) ketamine have been noted to have neurocognitive delays [2,3]. It has been impossible, to date, to determine if these neurocognitive delays are related to the ketamine, to the other anesthetic agents, or to a combination of both [3].

Currently, ketamine is being investigated for control of chronic pain [4,5] and for treatment of severe depression [6]. These indications necessarily require prolonged ketamine exposure compared to that required for induction of anesthesia or for sedation through a procedure.

If ketamine is going to be further developed as a medication with prolonged exposures, it is important to have an understanding of the neurocognitive toxicities of ketamine. In light of the above-discussed risks for ketamine exposure in children, this study used dosages of ketamine below anesthetic levels. Herein we report data obtained on neurocognitive function in children aged 11–19 with chronic pain exposed to daily, oral ketamine for 14 days.

## Methods

This study was approved by the Research Subjects Review Board of the University of Rochester and all participants completed an informed consent process and provided signed consent for their participation; parents provided written permission for the participation of minors (less than 18 years old). The study was registered on ClinicalTrials.gov before the first participant was enrolled (NCT01369680). The study conformed to the Food and Drug Administration guidelines for human subject’s protection (IND No. 110,951). Inclusion criteria were: Numerical Rating Scale (NRS) pain score ≥4, chronic pain for ≥3 months or persisting longer than expected for the underlying diagnosis, and age 8–22 years. The NRS pain had to be reported at least a 4 on the day of enrollment to meet eligibility criteria. This was reported by circling the number of “today’s pain” on a 10 cm NRS-11 pain scale [7]. The rationale for inclusion of children and adolescents aged 8–22 years old in this study was that they are still undergoing brain development. If this age group had notable lasting neurocognitive decline after daily exposure to low-dose ketamine, it would be reasonable to assume that younger children would be similarly harmed by daily exposure to low-dose ketamine. Chronic pain could be either idiopathic or related to a known diagnosis, including but not limited to cancer, rheumatologic disease, sickle cell anemia, cystic fibrosis, pancreatitis, and neuromuscular disease. Participants were ineligible for the study if there was a known or suspected history of: drug dependence or addiction, psychiatric disorder (depression, schizophrenia, bipolar disorder), or other medical problems thought to be unsafe during ketamine exposure. Participants were referred from pediatric services including neurology, orthopedic surgery, gastroenterology, rheumatology, and palliative care for participation in this clinical trial.

Full details of the dose-escalation protocol and rationale, eligibility criteria, and preparation and delivery of oral ketamine have been previously published [4]; the current report extends this work with special focus on the intermediate-term neurocognitive safety data. The study included 4 cohorts of 3 participants each, who were given dosages of oral ketamine of 0.25, 0.5, 1, or 1.5 mg/kg/dose three times a day. Participants in each cohort completed neurocognitive testing before, immediately after and 3 months after exposure to oral ketamine. Oral ketamine was administered three times per day for 14 consecutive days.

### Neurocognitive measures

The study was principally concerned with investigating safety and tolerability of ketamine, including potential cognitive effects of the drug. Therefore, participants completed a neurocognitive test battery that included assessments of attention and processing speed, memory, and executive function. The tests were selected because of prior literature [8–12] that suggested that these cognitive domains might be affected by exposure to ketamine.

Neurocognitive assessment was completed three times: at Baseline (prior to, but on the same day as, the first dose of ketamine), at Week 2 (between 24–48 hours after the final dose of ketamine), and at Week 14 (3 months after the final dose of ketamine). None of the participants took ketamine within 24 hours of the neurocognitive testing.

Most tests were drawn from CogState [13], a computerad-ministered, repeatable battery of neuropsychological tests. These tests were selected because they are brief, resistant to practice effects, and have well-established reliability and validity for the repeated assessment of cognitive function in children [14–16]. The entire battery took approximately 30 minutes for most participants and was administered in a quiet, private room by the study team. The CogState tasks are computer-administered; each task is preceded by scripted, written instructions that are also presented on the computer screen. Study team personnel read these standardized Cog State instructions to each participant for each testing session, unless the participant elected to read the directions on their own. Parents, family members, and friends were asked to step out of the private room for the duration of testing. CogState tasks and variables were chosen to evaluate selected cognitive domains previously suggested to be affected by ketamine exposure and are fully described in Appendix 1.

In addition to CogState, participants completed verbal fluency tasks to assess fluency for phonemic (first letter) and semantic categories. These tasks required participants to generate words as quickly as possible within 60-second trials. The phonemic fluency task utilized three trials, each requiring participants to generate words beginning with a specific letter of the alphabet (Controlled Oral Word Association; F-A-S). The dependent variable was the total number of correct words generated across all three trials. The semantic (Category) fluency task required participants to generate words within two specific categories (animals; food/drinks). The dependent variable for Category fluency was the total number of correct words generated over these two trials.

In addition to the formal neurocognitive assessment, cognitive symptoms were also included among the possible adverse events (AEs) and dose limiting toxicities (DLTs) that could be experienced. AEs, DLTs, and compliance with study medication were monitored at each visit and also weekly by phone. Cognitive DLTs for which participants were specifically monitored included hallucinations, delirium, confusion, mania, anxiety, amnesia, insomnia, agitation, dizziness, and depressed level of consciousness.

### Statistical analysis

Boxplots were obtained and descriptive statistics generated to evaluate all neurocognitive outcome variables for normality and outliers. Formal analyses were performed using three composite outcomes based on the following neurocognitive domains: Processing Speed (Detection response speed, Identification response speed, Chase moves per section); Memory (ISL Task correct responses; ISL Recall correct responses); and Executive Function (One Back response speed, Set Shifting accuracy, Groton Maze Learning Test total errors, Controlled Oral Word Association Test words generated, Category Fluency Test words generated). To create the composite variables, each individual variable within a cognitive domain at each study visit was transformed to a z-score using the mean and standard deviation from the baseline study visit to allow comparison of scores with different normal distributions. The assumption of normality was made for these analyses. These z-scores were then averaged across tests, resulting in a single composite score for Processing Speed, Memory, and Executive Function for each participant at each study visit. The null hypothesis tested was that there would be no decline in neurocognitive composite scores on average at Week 2 or Week 14. Formal analyses used a linear mixed model with time (visit week) as the independent variable (categorical) and neurocognitive domain score as the dependent variable. This model uses a direct-likelihood approach to accommodate missing data that is valid under the “missing at random” assumption [17]. The comparisons of primary interest were those representing differences between Baseline and Week 2 and between Baseline and Week 14. For each composite score, the magnitudes of the mean differences between Baseline and Week 2 and between Baseline and Week 14 were expressed using Cohen’s d [18]; this measure of effect size is defined as the mean difference divided by the residual standard deviation from the linear mixed model. All statistical analyses were performed with SPSS (version 20). No power analysis was performed for this study, as it was a pilot study.

## Results

### Participant details

Participants were aged 11–19 years (mean=16 years) and reported abdominal pain (n=3), joint or musculoskeletal pain (n=7), headaches (n=3), or allodynia (n=1). Participants did occasionally endorse pain in multiple sites and some had idiopathic musculoskeletal pain.

### Sample size and follow-up

In the first two cohorts (0.25 mg/kg and 0.50 mg/kg), 100% of participants (3 per group) were present for all three neurocognitive assessments. However, there was one non-completion for the Groton Maze Chase Test (GMCT) and the International Shopping List Test (ISRL) at Baseline do to computer malfunction. In the third dosage cohort (1.0 mg/kg), one participant did not complete the Week 14 evaluation due to transportation difficulties. In the fourth cohort (1.5 mg/kg), two of three participants were not evaluated at Week 14. Unwillingness to return for evaluations was thought to be related to these participants’ experience of adverse events (see below). In addition, the reliability of data obtained from one participant was deemed questionable due to motor and speech disabilities. Consequently, all data for this participant were removed from all analyses. Thus, the final sample size for neurocognitive assessment was N=11 participants (Table 1), though all 12 enrolled and treated participants are included in discussion of adverse events and pain scores.

### Neurocognitive assessment

**Figure 1:**
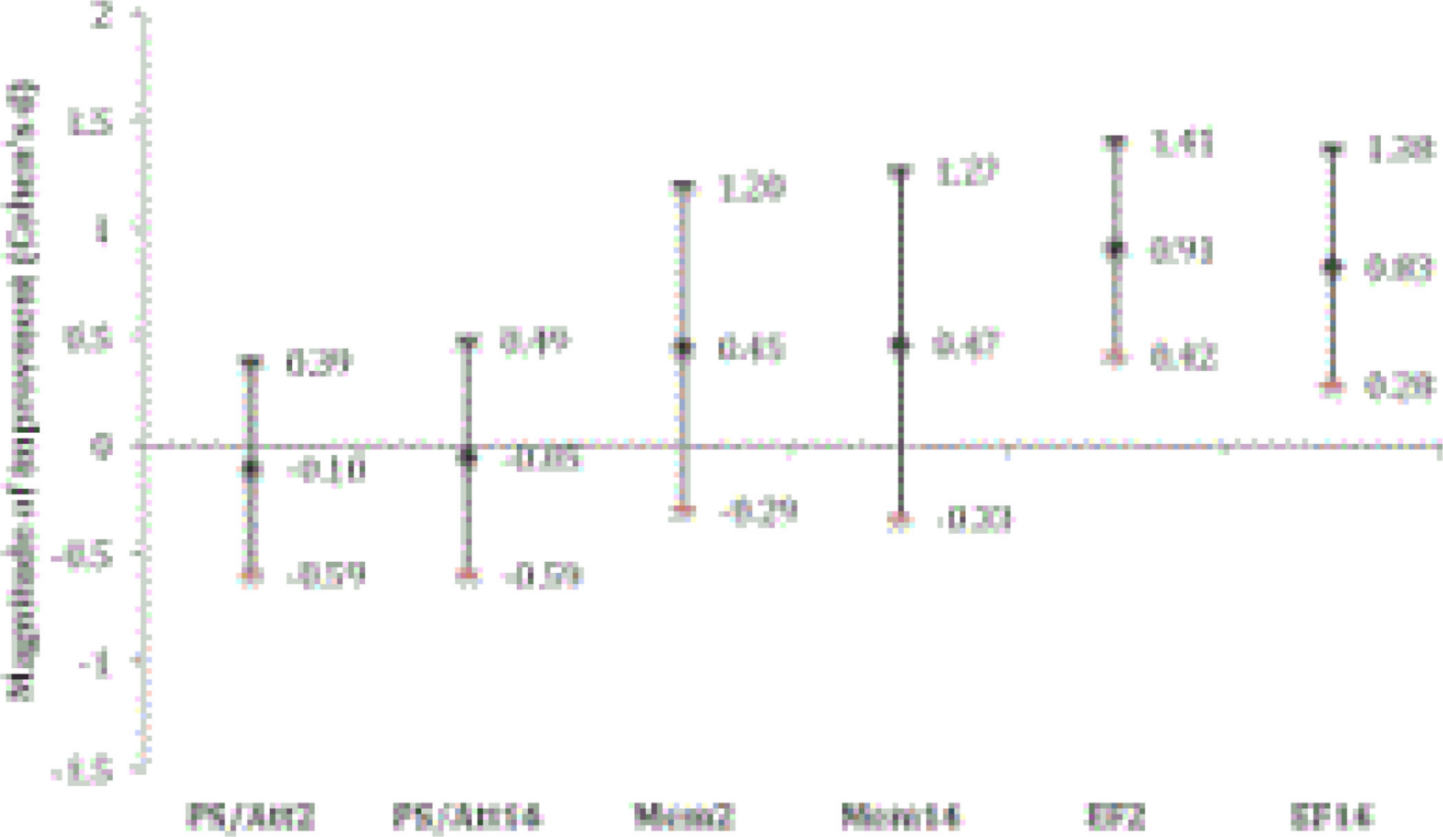
Magnitude of the improvement in performance between baseline and Weeks 2 and 14 on composite scores (Processing Speed/Attention, Memory, and Executive Function). Magnitude of improvement in children with chronic pain following a two-week course of ketamine. The 0 line represents performance at Baseline. Error bars represent 95% confidence intervals. Ps/Att2: Processing Speed/Attention Composite score at Week 2; Ps/Att14: Processing Speed/Attention Composite score at Week 14; Mem2: Memory Composite score at Week 2; Mem14: Memory Composite score at Week 14; EF2: Executive Function Composite score at Week 2; EF14: Executive Function Composite score at Week 14.

Summary longitudinal data for all neurocognitive outcomes are provided in Table 2. Linear mixed model analyses indicated that there were significant main effects of time on the Executive Function Composite (F(2,18)=8.66, p=0.002) but no significant main effects of time on the Processing Speed Composite (F(2,18)=0.10, p=0.91) or the Memory Composite (F(2,18)=1.06, p=0.37). The results of the planned comparisons (Week 2 vs. Baseline and Week 14 vs. Baseline), which are summarized in Figure 1, indicate that mean composite scores at Week 2 and Week 14 were not statistically different from those at Baseline for either the Processing Speed domain (Week 2: p=0.67, d (Cohen’s d)=−0.10; Week 14: p=0.84, d= −0.05) or the Memory domain (Week 2: p=0.22, d=0.45; Week 14: p=0.23, d=0.47). Significant mean improvements in the Executive Function Composite score were observed at Week 2 (p=0.001, d=0.91) and Week 14 (p=0.005, d=0.83). Improvements in Executive Function Composite scores at both Week 2 and Week 14 were noted.

### Neurocognitive Adverse Events and Dose Limiting Toxicities

Eleven of 12 participants in the study experienced adverse events while on study drug. Three participants discontinued participation prior to Week 14, all of whom discontinued study drug, due to an adverse event (new pain, decreased level of consciousness, and anorexia) prior to Week 2.

Two of these three adverse events were considered to be dose-limiting toxicities, both of which resolved within 2 days of discontinuing ketamine. Grade 1 adverse events involving neurologic changes included dizziness (N=4 participants) and confusion (N=4 participants). One participant experienced memory impairment (Grade 1 AE), and one experienced the dose limiting toxicity (Grade 2 AE) of depressed level of consciousness (complete sedation), which lasted for less than one hour and completely resolved following cessation of ketamine. Other non-cognitive DLTs are described in Bredlau et al. [4].

### Pain scores

Of the 12 participants treated in this study, 2 had complete resolution of pain by Week 2. Three other participants had decreases of 2 or more points on the NRS pain scale at Week 2. One participant had an increase in pain, and the other six participants had no change in pain scores.

Three of the six participants who had not had any change in pain scores were the three who did not complete the two-weeks of oral ketamine due to dose-limiting toxicities [4]. Table 3 lists pain scores for individual participants before and after ketamine exposure, along with executive function composite scores at the same time points.

## Discussion

This article describes the neurocognitive changes in children with chronic pain who received 14 days of oral ketamine. No evidence of decline in neurocognitive abilities was observed in this small cohort of children. However, given that this was a preliminary uncontrolled study with a small sample size, it is possible that subtle neurocognitive deficits were missed. Interestingly, and unexpectedly, we observed a statistically significant improvement in executive functioning. However, when directly queried, neither any child nor their parents reported a noticeable change in the child’s executive functioning.

Intuitively, it seems possible that children with chronic pain could experience decrements in neurocognitive test performance that is improved with appropriate pain control, as manifested by the increased (improved) executive function test scores in the current sample. Although it is known that children with chronic pain experience academic disruption (e.g., absenteeism) [19,20], that in turn may impact learning and cognitive performance, there are no studies that have definitively established a direct causal association between chronic pain and cognitive test performance in this diverse pediatric population. A recent study reported that lower intellectual ability in childhood (age 11 years) independently predicted higher chronic widespread pain over 30 years later, but noted that this relationship was mediated by both physiological (body mass index) and sociological (socioeconomic status) factors. In addition, the authors noted that “the poorer cognitive performance often reported in people with chronic pain might… long predate the development of that pain” (p. 2342) [21]. Similarly, in studies of adults with chronic pain, the association between chronic pain and cognitive performance is not yet clear, and is potentially confounded by motor slowing, mood dysfunction, fatigue, and motivational issues [22,23].

While it is recognized that individuals with chronic pain may have difficulty, relative to those without chronic pain, on highly demanding attention or executive function tasks, performance on less cognitively demanding tasks is not impaired. Moreover, psychosocial factors, including psychological distress and educational level, may significantly mediate this relationship, as may a participant’s interest in secondary gain. In another investigation of the relationship between pain and cognition in adults, there was no association between pain ratings and performance on tests of working memory (an executive function skill) among individuals with chronic pain, nor among healthy volunteers exposed to a new pain event (cold presser task). In addition, in both groups, neuropsychological test performance was normal. By contrast, among healthy volunteers who were instructed to malinger, or among chronic pain patients with a known history of malingering, cognitive test performance was impaired. While the authors noted that chronic pain might disrupt attentional control, they also reported that fewer than 5% of the non-malingering, chronic pain participants obtained cognitive test scores in the impaired range [24].

Given the previous literature on ketamine abuse, it is surprising that these children had an improvement in executive functioning after ketamine use. In fact, ketamine abuse (in adults with chronic, high-dose ketamine exposure) has been shown to be deleterious for executive functioning [9]. It is important to note, however, that these studies involved transiently induced pain in adults and were not “realworld” studies of children with chronic pain. In addition, these studies often examined the cognitive performance of participants during the active high-dose drug state. We are reassured that ketamine exposure at the dosages and delivery method used in the current study did not appear to have a sustained negative effect on cognition. Furthermore, consistent with prior studies, pain relief may improve cognitive control and/or reduce psychosocial distress, which is also implicated in the relationship between cognition and pain, as noted above.

Most of the participants in this study were not taking other medications that could affect their cognition, including executive function. Given that most of these children had chronic pain that was not responsive to medications; most of them were not taking opioid pain medications. Other medications to which the participants were exposed are not associated with changes in executive function [4] and were stable in dosage during the 2 weeks of oral ketamine exposure and cognitive function testing. The one exception was Participant 1001, who was on daily extended-release morphine, which provided minimal pain control prior to study participation. The participant’s pain was completely resolved on ketamine, and morphine was stopped on day 3 to avoid sedation from morphine while exposed to oral ketamine. A methadone taper was begun, due to the participant’s long-term exposure to morphine.

The small sample in this investigation is a limitation that might have diminished the ability to detect subtle neurocognitive change in participants. The sample was further reduced due to dropout of one subject who experienced ketamine toxicity. This was a transient, fully-reversible, dose-related effect (sedation). In retrospect it would have been interesting to pursue further cognitive assessment in this participant to ascertain whether or not neurocognitive changes occurred following cessation of ketamine. Another important limitation is the use of a non-randomized, uncontrolled design. The improvement in executive functioning scores could be spurious due to practice effects (i.e., improvement due to repeat exposure(s) to a task over a short period of time). Although the CogState tasks selected for this study were designed to minimize practice effects [16,25] it is possible that the Executive Function Composite score improved as a result of such effects, particularly in light of the inclusion of paper and pencil tests (COWAT and CFT) in the composite score [26,27]. It is difficult to evaluate this possibility, however, in the absence of a control group. In addition, the small sample size per dose group and lack of randomization precluded our ability to evaluate potential dose-response relationships.

The cognitive test data in this study were obtained principally as a component of secondary safety monitoring for the preliminary dosage-ranging trial of ketamine. The results will need to be verified with a larger sample size in a randomized, controlled trial before conclusions regarding the effects of exposure to 14 days of oral ketamine in children with chronic pain can be reached. In addition, those children experiencing dose-limiting toxicities were not available for Week 14 testing, yet they could have been the participants most likely to experience lasting neurocognitive decline on the study. Future studies will need to pay close attention to retention of participants, especially those experiencing dose-limiting toxicities.

The fact that participants did not complete cognitive testing during active ketamine exposure could also be considered a limitation. This study instead evaluated whether there were carryover neurocognitive changes following relatively short- and long-term discontinuation of ketamine. However, this approach can be viewed as providing more clinically relevant information, as the model for our ketamine therapy trial was a short-term (2 week) exposure for the treatment of chronic pain, as this is thought to be a sufficient duration for ketamine therapy in pediatric chronic pain [28,29]. It is encouraging that there were no detectable long-term effects of ketamine (3 months after its discontinuation) on cognition.

It is also possible that long-term neurocognitive decline may not be detectable in this study sample after only 3 months off therapy. However, given retention problems as outlined above, we thought it most reasonable to include assessments after a relatively short duration.

On the basis of prior preclinical and preliminary clinical findings, the primary concern for administering low dosage ketamine to children has been the possibility of a resulting decrement in neurocognitive function. The data presented here do not support the hypothesis that oral ketamine administered three times daily at low dosages for 14 days results in a sustained decrement in neurocognitive function, though participants did experience transient neurocognitive or neurologic adverse events while actively exposed to ketamine [4]. It is intriguing that executive function scores improved after administration of oral ketamine; however, this is in contradiction to previous data in ketamine drug abusers [8,10] and is not consistent with the preclinical data [30,31]. Data from randomized, controlled clinical trials are necessary to further characterize the impact (if any) of oral ketamine in children with chronic pain.

## Supplementary Material

Appendix

## Figures and Tables

**Table 1:** Baseline demographic characteristics of all participants contributing to neurocognitive data. (Modified with permission)

**Age (years)**	
Range	11–19
Median	16
**Sex**	
Female	8
Male	3
**Race**	
African-American	1
Caucasian	9
More than One Race	1 (White and African-American)
**Ethnicity**	
Hispanic	1
Not Hispanic	10

**Table 2:** Summary of tasks, associated outcome measures and cognitive domains, and scores at baseline, 2 weeks and 14 weeks;

Task	Measure	Cognitive Domain	Baseline (mean, SD)	Week 2 (mean, SD)	Week 14 (mean, SD)
			n=11	n=11	n=9
DET	Speed, log10ms	Processing Speed-Attention	2.46 (.06)	2.48 (.06)	2.42 (.17)
IDN	Speed, log10ms	Processing Speed-Attention	2.68 (.06)	2.70 (.08)	2.74 (.12)
GMCT	Moves per second	Processing Speed-Attention	1.38 (.40)*	1.52 (.31)	1.53 (.28)
ISLT	Total words recalled	Memory	25.90 (2.77)	28.55 (2.77)	27.89 (4.14)
ISRL	Total words recalled	Memory	9.80 (1.23)*	9.73 (1.62)	10.22 (1.56)
ONB	Speed, log10ms	Executive Function	2.93 (.06)	2.85 (.06)	2.87 (.08)
SETS	Accuracy, arcsine proportion correct	Executive Function	1.12 (.11)	1.18 (.08)	1.15 (.11)
GMLT	Total errors	Executive Function	49.73 (12.87)	41.55 (7.53)	39.00 (12.76)
COWA	# words generated	Executive Function	30.55 (8.50)	34.64 (9.12)	40.14 (13.42)
Category Fluency	# words generated	Executive Function	37.27 (9.93)	39.73 (8.97)	39.57 (13.84)

DET: Detection Task; IDN: Identification Task; GMCT: Groton Maze Chase Test; ISLT: International Shopping List Test, Immediate Recall; ISRL: International Shopping List Test, Delayed Recall; ONB: One Back task; SETS: Set Shifting task; GMLT: Groton Maze Learning Test; COWA: Controlled Oral Word Association test.

Note:

* There was one non-completion for the GMCT and the ISRL at Baseline.

**Table 3:** Summary of tasks, associated outcome measures and cognitive domains, and scores at baseline, 2 weeks and 14 weeks;

Participants	Baseline EC (z-score)	Baseline NRS pain score	Week 2 EC (z-score)	Week 2 NRS pain score
1001	−.238	5	.236	0
1002	.030	9	.934	7
1003	.364	10	.916	8
1004	.630	5	1.474	5
1005	−.960	4	−.202	9
1006	.392	5	1.316	5
1007	.438	4	1.008	0
1008	−.102	5	−.042	1
1009	−.018	6	1.044	6
1010	−1.092	0	−.268	0
1011	.338	6	.664	6

EC: Executive Composite score; NRS: Numerical rating scale (0=no pain, 10-worst imaginable pain).
