# Supplementary material for: Neurocognitive Changes after Sustained Ketamine Administration in Children with Chronic Pain
Source: J Palliat Care Med. Author manuscript; Available in PMC 2016 Mar 1. (PMC4618408; doi:10.4172/2165-7386.1000215)
Supplement: Appendix [file NIHMS714386-supplement-Appendix.pdf]

Appendix 1: Description of CogState tasks and variables

a) CogState Detection Task (DET): The Detection task is a measure of information processing speed and uses a well-validated simple reaction time paradigm using playing card stimuli. In this task, the playing cards are all red and black jokers. The participant is asked to press a "*Yes"* key as soon as the card in the center of the screen flips over. The dependent variable is performance speed, defined for this task as the mean of the $\log_{10}$ transformed reaction times for correct responses. Lower transformed scores indicate better (i.e., faster) performance.

b) Cog State Identification Task (IDN)**:** The Identification task is a measure of visual attention and uses a well-validated choice reaction time paradigm using playing card stimuli. In this task, the playing cards are all either red or black jokers. The participant is asked whether the card currently being presented in the center of the screen is red. The participant responds by pressing a "*Yes"* key when the joker card is red and "*No*" when it is black. The dependent variable is performance speed, defined as the mean of the $\log_{10}$ transformed reaction times for correct responses. Lower transformed scores indicate better (i.e., faster) performance.

c) CogState One Back Task (ONB): The One Back Memory Task is a measure of working memory and uses a well-validated n-back paradigm using playing card stimuli. In this task, the playing cards are identical to those found in a deck of playing cards with the exception of the joker. The participant is asked whether the card currently shown is the same as the preceding card. The participant responds by pressing *Yes* or *No* keys. The first response is always treated as *No,* since there is no preceding card for comparison. The primary dependent variable is performance speed, mathematically defined as the mean of the $\log_{10}$ transformed reaction times for correct responses. Lower transformed scores indicate better (i.e., faster) performance.

d) CogState Set Shifting Task (SETS)**:** The Set Shifting Task is a measure of executive function. In this task, playing cards are presented on the screen one at a time. Participants are required to determine if each playing card is 'correct' or 'incorrect', selecting *Yes* or *No* keys, respectively. These decisions are based on underlying rule sets related to either the color of the card (red or black) or the number shown on the card. Participants learn test rules through trial-and-error strategies, and by using feedback from the computer; when a wrong choice is made, an error tone is sounded and the participant must correct his or her response in order for the test to proceed. In addition, test rules change over time at a general level (whether color or number is the rule), and at a more specific level (which color or number is correct, within the respective general rule). The dependent variable is performance accuracy, defined for this task as the mean of the arcsine transformed proportion of correct responses; higher scores indicate better performance.

e) Groton Maze Chase Test (GMCT); Groton Maze Learning Test (GMLT): The *Groton Maze Chase Test* is a measure of visuomotor speed. In the Chase task, participants follow a bulls-eye target around a 10 x 10 square grid displayed on the computer screen, observing specific rules for moving across the grid (e.g., do not skip tiles, no diagonal moves). For this task, the dependent measure reflects the

combination of response speed and accuracy, defined as the total number of correct moves per second (higher scores indicate better performance). The Chase task also provides training on the rules for the ***Groton Maze Learning Test*,** which is a measure of problem solving and reasoning and uses a well-validated maze-learning paradigm. As with the Chase task, ***GMLT*** presents participants with a 10 x 10 grid of squares. Participants are required to learn a 28-step pathway hidden within the grid, again guided by similar rules as for the Chase task. Correct choices are rewarded with a green check mark and the ability to continue learning subsequent steps of the maze. Incorrect choices require the participant to return to the last correct move and explore other options to learn the path. Thus, through trial-and-error learning, participants identify the path. Participants repeat the same path several times in succession using the same trial-and-error learning approach and are expected to complete these subsequent trials ever more efficiently as they learn the path. The primary dependent variable is a measure of accuracy, defined as the total number of errors across all learning trials.

***f) CogState International Shopping List Task (ISLT); International Shopping List Recall (ISLR)*:** The International Shopping List task is a measure of rote verbal learning and memory and uses a well-validated list-learning paradigm. High frequency, high imagery concrete nouns (items from a shopping list) are read to the participant by the test supervisor at the rate of one word every two seconds. The list is presented three times; after each presentation the participant is asked to recall as many of the words as he or she can, as quickly as possible. Accurate responses are recorded on the computer screen by the test supervisor examiner. The dependent variable is the total number of correct responses across all three learning trials. The ISL Recall trial evaluates delayed recall for the original shopping list words. Participants are required to recall the words from the list 15-30 minutes after their initial presentation, without additional presentations of the list. The dependent variable is the total number of correct responses on the delayed recall trial.
